# Characterization and Phylogenetic Analysis of Ancient Italian Landraces of Pear

**DOI:** 10.3389/fpls.2017.00751

**Published:** 2017-05-10

**Authors:** Nicoletta Ferradini, Hovirag Lancioni, Renzo Torricelli, Luigi Russi, Isabella Dalla Ragione, Irene Cardinali, Gianpiero Marconi, Mauro Gramaccia, Luciano Concezzi, Alessandro Achilli, Fabio Veronesi, Emidio Albertini

**Affiliations:** ^1^Dipartimento di Scienze Agrarie, Alimentari e Ambientali, Università degli Studi di PerugiaPerugia, Italy; ^2^Dipartimento di Chimica, Biologia e Biotecnologie, Università degli Studi di PerugiaPerugia, Italy; ^3^3A Parco Tecnologico Agroalimentare Dell'Umbria – TodiPerugia, Italy; ^4^Dipartimento di Biologia e Biotecnologie “L. Spallanzani”, Università di PaviaPavia, Italy

**Keywords:** *Pyrus* spp., SSR markers, chloroplast DNA, hypervariable intergenic region, local varieties, phylogeny, genetic resources

## Abstract

Pear is one of the oldest fruit tree crops and the third most important temperate fruit species. Its domestication took place independently in the Far East (China) and in the Caucasus region. While the origin of Eastern Asian cultivars is clear, that of European cultivars is still in doubt. Italy has a wealth of local varieties and genetic resources safeguarded by several public and private collections to face the erosion caused by the introduction of improved varieties in specialized orchards. The objectives of the present study were: (i) to characterize the existing germplasm through nuclear (SSR) and (ii) to clarify the genetic divergence between local and cultivated populations through chloroplast DNA (cpDNA) markers in order to provide insights into phylogenetic relationships of *Pyrus* spp. For this reason, 95 entries from five different germplasm collections, including nine European, Mediterranean and Eastern Asian species, were analyzed, and the intergenic *accD-psaI* sequences were compared to the worldwide distributed dataset encompassing a total of 298 sequences from 26 different *Pyrus* species. The nine nuclear SSRs were able to identify a total of 179 alleles, with a *loci* polymorphism *P* = 0.89. Most of the variation (97%) was found within groups. Five accessions from different sources were confirmed to be the same. Eight out of 20 accessions of unknown origin were identified, and six synonyms were detected. *Locus* NH030a was found to be monomorphic in all the cultivated accessions and in reference species interfertile with *P. communis*, leading to hypothesize selection pressures for adaptation to cultivation. The cpDNA sequences of the 95 accessions were represented by 14 haplotypes, six of which (derived from *P. communis, P. cossonii* and *P. ussuriensis*) are recorded here for the first time and may suggest the ancient origin of some local varieties. The network analysis of the 298 cpDNA sequences allowed two different haplogroups, Eastern and Western Eurasia, to be defined, supporting recent views of a clear division between Occidental and Oriental species. By combining the results from nuclear and uniparental markers, it was possible to better define many unknown accessions.

## Introduction

Pear belongs to the Rosaceae family, the Maloideae subfamily, and is the third most important temperate fruit species after grape and apple. It is widespread throughout the world with China, the United States, Italy, Argentina, and Spain being the most important producers. Annual pear production in the world is about 25 million tons, 3% of which is produced in Italy, making it the first European producer (FAO, 2014).

The genus *Pyrus* is characterized by a high genetic variability with several species and thousands of cultivars that can be divided into two major groups, the Occidental (European) and the Oriental (Asian) pears. Being cultivated for more than 3,000 years, pear is also one of the oldest fruit crops in the world (Wu et al., 2013, 2014). The primary centers of origin and of domestication of the genus *Pyrus* are China and the region from Asia Minor to the Middle East, in the Caucasus Mountains, while a secondary center is located in Central Asia (Vavilov, 1951; Zukovskij, 1962; Silva et al., 2014). Even if the most cultivated species are *P. ussuriensis, P. pyrifolia, P. bretschneideri, P. communis*, and *P. sinkiangensis*, to date as many as 22 species have been well recognized (Wu et al., 2013). The basic chromosome number of the Maloideae subfamily is *x* = 17, a significantly higher number than that of other Rosaceae species (*x* = 7 or *x* = 9), thus suggesting a possible polyploidization event (Sax, 1931). According to Silva et al. (2014), the *Pyrus* genus could be the result of a hybridization between two primitive forms of Rosaceae: Prunoideae (*x* = 8) and Spiraeodeae (*x* = 9). The majority of cultivated pears are diploid (2n = 2x = 34), but a few cultivars of *P. communis* and *Pyrus* × *bretschneideri* are also known to be polyploids. It is also believed that cultivated European pears derive from two wild pears, *P. pyraster* and *P. caucasica*, which are interfertile with domesticated forms (Zohary and Hopf, 2000).

Despite its presence in Europe from prehistoric time and its economic importance, the exact origin of the European pear is still unknown; on the contrary, places and times of domestication for Asian pears are clearer (Silva et al., 2014).

From the historical point of view, Homer was the first to mention pear cultivation, followed by Marco Porcio Catone (253–150 BC), Varrone, Virgilio, Celso, and by Plinius the Elder, who described almost all of the 40 varieties present in the Roman Empire at their times (Hedrick, 1921; Layne and Quamme, 1975; Columella, 1977; Silva et al., 2014). In the Italian peninsula, after the fall of the Roman Empire, agriculture underwent intense changes, accompanied by modification of the landscape (Andreolli, 1990). In such a context, pears were basically cultivated only in vegetable gardens, mainly for family consumption, honey production and quality wood (Gaulin, 2007). In the Middle Ages the Benedictine monks played a major role in selection, conservation and cultivation of pear varieties, role maintained also in the following period (XVI century) when métayage contracts contributed to increasing the diversification of cultivation (Anselmi, 2000). Starting from the XVIII century many varieties were systematically introduced into Central Italy from other Italian areas, as well as from foreign countries, as documented by botanists and agronomists of that period, such as Felici (1565), Durante (1585), and Caporali (1599). The presence of French/Belgian (Decana del Comizio, Abate Fetel, Curato, Bergamotte Esperen, Passa Crassana) and English (William) varieties was historically well documented and widespread in many other areas, increasing pear diversity and most likely changing of their names. In more recent decades, the introduction of modern and more productive pear cultivars grown in specialized orchards has caused a drastic decrease in diversity and the genetic erosion of ancient cultivars.

Assessment of the existing genetic diversity is a preliminary step in order to plan for its safeguard and its use in breeding programs. Several studies based on molecular markers have estimated the diversity in *Pyrus*, including modern cultivars and germplasm accessions. RFLPs (Iketani et al., 1998), RAPDs (Oliveira et al., 1999; Teng et al., 2001, 2002), AFLPs (Monte-Corvo et al., 2000; Dolatowski et al., 2004; Bao et al., 2008) and SSRs (Kimura et al., 2002; Bao et al., 2007; Wünsch and Hormaza, 2007; Miranda et al., 2010; Gasi et al., 2013; Akçay et al., 2014; Zheng et al., 2014; Urrestarazu et al., 2015) have been widely used to estimate the genetic diversity and the relationships between pear cultivars, wild forms, and related species. Some ancient Italian pear accessions have also been characterized using morphological and SSR markers (Martinelli et al., 2009).

Recently, in order to reconstruct the phylogeny and the evolution of several genera, scientists have directed their attention to the variability of the chloroplast DNA (cpDNA) (Crosby and Smith, 2012; Vieira et al., 2014; Pervaiz et al., 2015). Due to its peculiarities, such as uniparental inheritance, absence of recombination and high level of genetic diversity, cpDNA analyses are very useful for assessing the phylogenetic structure of different populations (Nock et al., 2011; Scarcelli et al., 2011; Yang et al., 2013; Wheeler et al., 2014). Noncoding DNA regions of cpDNA have been successfully applied to phylogenetic studies of plants at lower taxonomic levels, as in the Rosaceae taxa (Corriveau and Coleman, 1988), and in *Pyrus* in particular (Kimura et al., 2003; Katayama et al., 2007, 2012).

Moreover, uniparentally inherited genomes are sensitive to historical bottlenecks (Morgante et al., 1997) and cpDNA markers might provide some genetic information about a species. Therefore, a combined analysis of nuclear SSR markers and cpDNA haplotypes could provide a more comprehensive view of population structure and demographic history (Burban and Petit, 2003; Petit et al., 2005).

The present research aims to provide further insight into the phylogenetic relationships of *Pyrus*, starting from native local accessions across the entire genus. This comparative analysis involved 95 pear accessions (prevalently classified as *P. communis*, but also *P. pyraster, P. caucasica* and some Asian species), and all intergenic segment *accD-psaI* recorded in GenBank, encompassing a total of 298 sequences of 26 different *Pyrus* species from both cultivated and wild populations. Integrating nuclear and cpDNA markers, it thus represents one of the first comprehensive phylogenetic and phylogeographic analyses of different pear populations distributed from East to West in the Eurasian region. It is expected to shed light on the geographical origins and ancestral populations of cultivated pear species, in order to clarify the genetic divergence between local and cultivated populations and to define appropriate management of the local genetic resources.

## Materials and methods

### Plant material

Ninety-five accessions from European and Asiatic *Pyrus* spp. were included in this study (Table 1 and Table S1). Many of the 95 accessions were well documented by historically reliable sources. For others, lacking some information, the original name, like that given by the donor, was maintained. In all other cases they were named Unknown. Therefore, based on the initial information, the 95 accessions used in this study were grouped into reference species (RS), commercial varieties (CV), local varieties (LV) and unknown accessions (UA), where CV and RS were used as controls. Moreover, 75 accessions were divided into three geographic groups, following the classification of Fideghelli (2007): Mediterranean (2), East-Asian (4), and European (69 in total: 24 CV, 2 RS, 43 LV). Because the remaining 20 accessions could not be assigned to any geographic group, they were grouped as Unknown (Table S1).

**Table 1:** **Names, status and codes of the 95 pear accessions used in the present study**.

Code^*^	**Accession name**	**Status**
gr1_001	Unknown	UA
gr1_002	Unknown	UA
gr1_004	Pera Trentonce	LV
gr1_005	Unknown	UA
gr1_006	Unknown	UA
gr1_007	Pera Agostina	LV
gr1_008	Unknown	UA
gr1_009	Pera Sementina	LV
gr1_010	Unknown	UA
gr1_011	Unknown	UA
gr1_012	Unknown	UA
gr1_013	Pera Grassana	LV
gr1_014	Pera Ruzza	LV
gr1_015	Pera Campana	LV
gr1_016	Pera Monteleone	LV
gr1_018	Pera Mezza	LV
gr1_020	Coscia	CV
gr1_021	Unknown	UA
gr1_022	Unknown	UA
gr1_023	Unknown	UA
gr1_024	Unknown	UA
gr1_025	Unknown	UA
gr1_027	Unknown	UA
gr1_028	Unknown	UA
gr1_029	Unknown	UA
gr1_030	Unknown	UA
gr2_032	Pera Ammazza Cavallo	LV
gr2_034	Bergamotte Esperen	CV
gr2_035	Pera Broccolina	LV
gr2_036	Scipiona	CV
gr2_037	Martin Sec	CV
gr2_038	Mora di Faenza	CV
gr2_039	Cedrata Romana	CV
gr2_040	Angelica	CV
gr3_042	Pera Volpina	LV
gr3_043	Pera Monteleone	LV
gr3_044	Unknown	UA
gr3_045	Pera Burro	LV
gr3_046	Pera della Trebbiatura	LV
gr3_047	Pera Tonda Roggia	LV
gr3_048	Pera Vernia	LV
gr3_049	Pera Prestareccia	LV
gr3_050	Spadona d'Inverno	CV
gr3_051	Pera Limoncina	LV
gr1_052	Pera San Pietro	LV
gr3_053	Unknown	UA
gr3_054	*P*. *pyraster*	RS
gr3_055	Pera Agostina	LV
gr3_056	Pera della Battitura	LV
gr3_058	Pera Limone	LV
gr3_059	Pera San Pietro	LV
gr1_061	Pera di Montelupone	LV
gr1_062	Madernassa	CV
gr1_063	Unknown	UA
gr4_065	Duchesse d'Angouleme	CV
gr4_066	Bergamotte Esperen	CV
gr4_067	*P. ussuriensis*	RS
gr4_068	Coscia Tardiva	CV
gr4_069	Carmen	CV
gr4_070	Conference	CV
gr4_071	Coscia Precoce	CV
gr4_073	Passa Crassana	CV
gr4_074	Angelica	CV
gr4_075	Decana del Comizio	CV
gr4_076	Pera Spadoncina Estiva	LV
gr4_077	William	CV
gr4_078	Kaiser	CV
gr4_079	Butirra Precoce Morettini	CV
gr4_080	*P. caucasica*	RS
gr4_081	Curato	CV
gr4_082	*P. pyrifolia*	RS
gr4_083	Guyot Precoce	CV
gr4_085	*P. calleryana*	RS
gr4_086	*P. betulifolia*	RS
gr4_087	Santa Maria Morettini	CV
gr4_088	*P. cossonii*	RS
gr4_089	*P. syriaca*	RS
gr5_090	Pera Fiorentina	LV
gr5_091	Pera Lardaia	LV
gr5_092	Pera Moscatella Tardiva	LV
gr5_093	Pera Cane	LV
gr5_094	Pera Grossa d'Autunno	LV
gr5_095	Pera Leccia	LV
gr5_096	Pera Rubbia	LV
gr5_097	Pera Lardaia	LV
gr5_098	Pera Marzola	LV
gr5_102	Pera Somentina	LV
gr5_103	Pera Bianchina	LV
gr5_104	Pera Volpina	LV
gr5_105	Pera Moscatella	LV
gr5_106	Pera Brutta Buona	LV
gr5_109	Pera Cannella	LV
gr5_110	Pera Garofina	LV
gr5_112	Pera di Tiberio	LV
gr5_117	Pera Briaca	LV

* *gr1 and gr3 were provided by 3A-PTA (Parco Tecnologico Agroalimentare dell'Umbria), gr2 by Adanti private collection, gr4 by CREA (National Centre of Fruit Tree Germplasm) and gr5 by “Archeologia Arborea” private collection*.

### Microsatellite amplification

Total genomic DNA was isolated from young leaves using the DNeasy Plant Kit (Qiagen) following the protocol provided by the manufacturer. Nine pear SSR primer combinations (Yamamoto et al., 2002a,b) were used (Table S2). PCRs were carried out with the Type-it Microsatellite PCR Kit (Qiagen) containing 1X Type-it master mix with 0.2 μM of each forward and reverse primer and 20 ng of DNA and H_2_O to a final volume of 20 μl. Amplification was performed as follow: an initial step at 95°C for 5 min followed by 30 cycles at 95°C for 30 s, 54–62°C for 30 s, and 72°C for 30 s, and a final extension at 72°C for 10 min.

PCR products were separated and analyzed on a 3130 XL DNA Analyzer (Applied Biosystems). The size of the amplified products was determined on internal standard DNA (GeneScan 500 Liz, Thermo Fischer Scientific) and the scorable peaks were assigned by GeneMapper software (Applied Biosystems).

### Chloroplast non-coding region amplification

The hypervariable *accD-psaI* intergenic spacer was amplified by PCR using *accD* F2 (Zheng et al., 2014) and *psaI* 75R (Small et al., 1998) primers. PCR reactions contained: 1X Phusion HF Buffer, 200 μM dNTPs each, 0.5 μM each primer, 0.2 U Phusion *Taq* DNA polymerase (Thermo Fischer Scientific), 30 ng genomic DNA and H_2_O to a final volume of 50 μl. PCR amplification was carried out by GeneAmp PCR system 9700 (Applied Biosystems) programmed as follow: 98°C for 30 s, followed by 30 cycles of 98°C for 10 s, 66°C for 15 s, 72°C for 30 s, and then 72°C for 10 min.

The purified PCR products of about 1,000 bp were sequenced at the *Polo di Innovazione Genomica, Genetica e Biologia* (Perugia, Italy). The new cpDNA sequences were recorded in GenBank with accession numbers from KY606436-KY606530.

### Data analysis

The statistical analysis of the SSR data matrix included (i) the estimation of observed (*Ho*) and expected (*He*) heterozygosity (Nei, 1978), (ii) the F statistics (F_is_ and F_st_) (Weir and Cockerham, 1984), and (iii) the analysis of molecular variance (AMOVA) by estimating the fraction of the genetic variation among and within populations (Excoffier et al., 1992; Michalakis and Excoffier, 1996). The software packages GENODIVE (Meirmans and Van Tienderen, 2004) and SPAGeDi1.2 (Hardy and Vekemans, 2002) were used for these purposes, being able to analyze data files containing diploid and triploid accessions.

SSR data were also converted to a binary data matrix by assigning “0” to the absence of a defined allele and “1” to its presence, and were used to estimate a similarity matrix using the coefficient of Dice (Dice, 1945), and the individuals were clustered by the unweighted pair group method with arithmetic mean (UPGMA) and validated by 1,000 bootstrap replicates using PAST software (Hammer et al., 2001).

The SSR profiles of the 95 accessions were used to investigate the population structure through the Bayesian model-based clustering procedure of STRUCTURE ver. 2.2.3 (Pritchard et al., 2000). The analyses were based on an admixture ancestral model with correlated allele frequencies, and the number of *K* clusters was determined by simulating a range of *K*-values starting from one to ten. A burn-in and a run length of the Monte Carlo Markov Chain (MCMC) of 200,000 and 500,000 iterations for data collection with 10 runs per *K*-value were used. The best *K*-value was determined through the Δ*K* method (Evanno et al., 2005) by using Structure Harvester ver. 0.6.193 application (Earl and vonHoldt, 2012). A second step analysis of STRUCTURE was then performed separately at these *K*-values with 600,000 burning period and 1,000,000 MCMC repeats after burning. The 95 individuals were assigned to the groups according to their highest membership coefficient, considering a strong affinity when the assigning probability (*qI*) was ≥0.80 (Breton et al., 2008; Pereira-Lorenzo et al., 2008; Miranda et al., 2010; Urrestarazu et al., 2012).

In order to analyze the cpDNA sequences, forward and reverse sequences from each sample were assembled and aligned using Sequencher™ 5.10 (Gene Codes Corporation). Accession gr4_077 *P. communis* cultivar William was selected as a reference sequence (GenBank accession number KY606501). It is one of the most common and suitable exemplars of European pear cultivars.

Different cpDNA sequence variation parameters were estimated by using DnaSP 5.1 software (Librado and Rozas, 2009). AMOVA was carried out as reported above for the SSR data. The 95 sequences were also compared to all *accD-psaI* data recorded in GenBank: 203 *Pyrus* sequences from 26 species and 4 putative inter-specific hybrids. The final dataset included 298 *Pyrus* accessions from three major geographic areas: Europe, the Mediterranean Area and Asia; the latter included Eastern and Middle Eastern subgroups (Tables S3, S4). A sequence from *Malus domestica* was included and used as an outgroup (Katayama et al., 2012). Indels of different lengths at the same position were separately treated and coded with the number of inserted or deleted nucleotides (Table S5). The final alignment was compared to the reference sequence, thus allowing sequence classification in different haplotypes. The evolutionary relationships among haplotypes were visualized through the construction of a Median-Joining network using the software Network 4.6 (http://www.fluxus-engineering.com/), each indel was considered as a single mutational event and partitioned by using different indel codes (Table S6).

As for principal component analysis (PCA), performed by XLSTAT (2011) statistical software, each species was considered as a discrete variable, the initial dataset was converted into principal components (PCs) and it was possible to graphically display the relationships among the intergenic regions *accD-psaI* of all sequences.

## Results

### Genetic diversity

Scorable amplicons were produced for all nine nuclear SSRs, with a total of 179 alleles. The average number of alleles per *locus* was 20, ranging from five (NH030a) to 29 (NH023a), but the number of effective alleles per *locus* was significantly lower (NAe = 6.8). At locus NH030a, out of five alleles, allele 167 showed a frequency of 0.97; thus *loci* polymorphism was *P* = 0.89 (Cavalli-Sforza and Bodmer, 1971).

As many as 24 individuals out of 95 (23%) showed only one *locus* with a third allele. Ten genotypes showed 2 *loci* with a third allele (9.5%), while nine individuals showed a third allele at more than 2 *loci* (Table S7). In particular, except for *locus* NH030a, all other *loci* showed at least one genotype with three alleles. NH026a and NH023a identified 16 and 18 individuals with three alleles, respectively; NH029a had only two, while the other *loci* identified between seven and ten individuals with three alleles.

The AMOVA carried out with all *loci* showed that most of the existing variability was within groups (97%) rather than among groups (Table S8), which is in agreement with many outbreeding species and similar studies in pear (Jiang et al., 2009; Miranda et al., 2010; Wolko et al., 2015; Wuyun et al., 2015). Nonetheless, differences among the 4 groups (CV, LV, RS, and UA) were found in terms of the effective number of alleles and levels of heterozygosity.

The 95 individuals in the present study were not a panmictic population, as classically defined in population genetics. Therefore, the indices used here (number of effective alleles, observed and expected heterozygosity, F_IS_ and F_ST_) as estimates of existing genetic variability should be considered with caution. It is worth noting that the effective number of alleles (NAe) is a measure of the genetic variability (Zouros, 1979), also valid in the presence of small samples (Nielsen et al., 2003). In our data the highest average number of effective alleles was found in RS (NAe = 12.4), while the lowest was in CV (4.8) (Table 2); it is interesting to note that the values of LV and UA were intermediate and similar to one another (6.9).

**Table 2:** **Range of SSR allele size per ***locus***, number of alleles (NA) and number of effective alleles per ***locus*** (NAe), expected (H_**e**_) and observed (H_**o**_) heterozygosity in all individuals and in each group based on available information, as estimated with SPAGeDi 1.5**.

**Locus**	**Size range (bp)**	**All groups (*****n*** = **95)**				**Reference species (*****n*** = **8)**				**Commercial varieties (*****n*** = **24)**				**Local varieties (*****n*** = **43)**				**Unknown accessions (*****n*** = **20)**			
		**NA**	**NAe**	**H_e_**	**H_o_**	**NA**	**NAe**	**H_e_**	**H_o_**	**NA**	**NAe**	**H_e_**	**H_o_**	**NA**	**NAe**	**H_e_**	**H_o_**	**NA**	**NAe**	**H_e_**	**H_o_**
NH019b	166–203	15	2.4	0.57	0.56	9	3.8	0.74	0.75	6	2.6	0.61	0.58	9	2.2	0.54	0.52	5	2.2	0.55	0.55
NH023a	106–192	26	4.0	0.75	0.57	10	8.7	0.89	0.50	6	3.2	0.69	0.67	17	3.8	0.74	0.55	13	4.6	0.78	0.50
NH026a	109–183	29	9.8	0.90	0.51	12	20.1	0.96	0.88	9	4.6	0.78	0.44	22	13.4	0.93	0.50	15	8.0	0.88	0.45
NH027a	113–182	26	10.6	0.91	0.85	12	19.1	0.96	0.75	11	7.2	0.86	0.83	17	10.3	0.90	0.88	13	9.6	0.90	0.85
NH029a	80–101	12	6.1	0.84	0.71	10	11.4	0.92	0.88	8	6.3	0.84	0.54	9	5.4	0.82	0.79	8	5.4	0.81	0.65
NH030a	167–205	5	1.1	0.06	0.04	5	2.5	0.61	0.50	1	1.0	0	0	1	1.0	0	0	1	1.0	0	0
Nb103a	72–122	22	8.1	0.88	0.91	12	21.3	0.96	0.75	11	6.1	0.84	0.92	16	6.9	0.85	0.91	12	9.8	0.90	0.95
Nb105a	140–194	18	8.0	0.88	0.91	12	7.6	0.87	0.88	10	5.2	0.81	0.83	12	8.3	0.88	0.93	12	9.4	0.90	0.95
Nb109a	122–188	26	11.1	0.91	0.92	11	17.3	0.95	0.75	12	6.7	0.85	1.00	20	11.1	0.91	0.88	15	12.0	0.92	0.95
All loci	–	19.9	6.8	0.74	0.66	10.3	12.4	0.87	0.74	8.2	4.8	0.70	0.65	13.7	6.9	0.73	0.66	10.4	6.9	0.74	0.65

A similar trend was observed in terms of heterozygosity. The average expected heterozygosity, considering all of the pear accessions, was *He* = 0.74, ranging from 0.87 in RS to 0.70 in CV. The highest mean expected value of heterozygosity for all accessions was found at *loci* Nb109a and NH027a (*He* = 0.91), while the lowest was at *locus* NH030a (*He* = 0.063). Noticeably, at this *locus, He* in the reference species was 0.61, while in all other groups (LV, CV, and UA) *He* = 0, monomorphic and due to the fixation of allele 167. In addition, in RS, NH026a, NH027a, and Nb103a *loci* showed *He*-values as high as 0.96 (Table 2).

The mean F_ST_-value equal to 0.014 (Table 3) indicates a moderate differentiation among the four groups (*P* < 0.0005), thus confirming that most of the variation is within groups; at *locus* NH030a the F_ST_ value of 0.278 indicates a significantly high genetic differentiation (*P* < 0.0002), suggesting that at this *locus* the selection for local adaptation could have been so strong as to restrict its variation to a single allele in all cultivated forms.

**Table 3:** **F-Statistics as estimated by SPAGeDi 1.5 (Weir and Cockerham, 1984) of 95 entries of ***Pyrus*** spp. grouped by Reference species (RS), Commercial varieties (CV), Local varieties (LV), and Unknown accessions (UA), and based on the 9 simple sequence repeat ***loci*** (alone and all together)**.

**Locus**	**All groups (*****n*** = **95)**				**RS (*****n*** = **8)**		**CV (*****n*** = **24)**		**LV (*****n*** = **43)**		**UA (*****n*** = **20)**	
	**F_IS_**	***P***	**F_ST_**	***P***	**F_IS_**	***P***	**F_IS_**	***P***	**F_IS_**	***P***	**F_IS_**	***P***
NH019b	−0.015	0.8805	−0.003	0.9344	−0.060	0.7185	−0.020	0.8892	0.019	0.7625	−0.021	0.9650
NH023a	0.174	0.0000	0.002	0.6428	0.404	0.0010	−0.018	0.8792	0.174	0.0031	0.275	0.0026
NH026a	0.352	0.0000	0.017	0.0381	0.093	0.3279	0.350	0.001	0.359	0.0000	0.439	0.0000
NH027a	0.039	0.2163	0.016	0.0257	0.198	0.0408	0.030	0.6504	0.009	0.7983	0.050	0.4363
NH029a	0.147	0.0012	0.005	0.5202	0.044	0.5606	0.338	0.0005	0.031	0.6419	0.206	0.0544
NH030a	0.145	0.3573	0.278	0.0002	0.188	0.3475	–	–	–	–	–	–
Nb103a	−0.049	0.1632	0.015	0.0295	0.203	0.0292	−0.099	0.2467	−0.071	0.1706	−0.059	0.482
Nb105a	−0.056	0.1237	0.017	0.0245	0.001	0.9235	−0.047	0.6417	−0.065	0.1984	−0.067	0.4053
Nb109a	−0.024	0.4452	0.015	0.0163	0.222	0.0554	−0.171	0.0056	0.025	0.5494	−0.044	0.4841
All *loci*	0.0734	0.0000	0.014	0.0005	0.148	0.0003	0.047	0.1233	0.063	0.0015	0.099	0.0019

### Genetic structure

**Figure 1:**
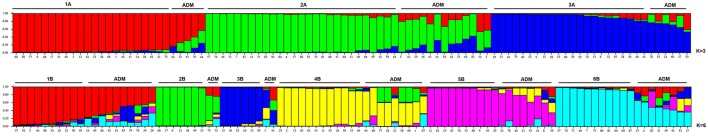
**Structure classification at ***K*** = 3 and ***K*** = 6 of the 95 entries of ***Pyrus*** spp**. The clusters are marked with different colors, while the accessions at *P* of (*qI*) ≤ 0.80 are grouped as Admixture (ADM).

The nine SSRs were also used to determine the genetic structure among the 95 accessions of *Pyrus* spp. The plot of the average log-likelihood values for *K*s ranging from 1 to 10 and the distribution of Δ*K*-values (Evanno et al., 2005) according to *K*-values is shown in Figure S1. Two peaks were found, corresponding to *K* = 3 and *K* = 6, and the hierarchical genetic structure was investigated at *K* = 3. A threshold value qI ≥ 0.80 was used to assign individuals to the clusters (Figure 1). Structure at *K* = 3 was able to define 3 clusters (from 1A to 3A). Cluster 1A included mostly commercial cultivars and *P. syriaca*, one of the reference species able to intercross with *P. communis*. Cluster 2A included 27 accessions, almost all the rest of the reference species (six, except *P. cossonii*), most of LV provided by the “Archeologia Arborea” collection and only one commercial cultivar (Carmen). Cluster 3A included 22 accessions, mostly LV (12) and 7 CV, four of which were of French origin. Some accessions (11 LV, 6 CV, 5 UA, and *P. cossonii*) were clustered in the admixture group. Although this was not the object of the present study, as physiological and phenotypic information was scarce and incomplete, Cluster 1A included the majority of accessions characterized by summer ripening, Cluster 2A all accessions with an autumn-winter ripening period, while accessions in Cluster 3A were not clear cut in terms of ripening period.

The structure at *K* = 6 allowed six Clusters (from 1B to 6B, Figure 1) to be distinguished. Cluster 1B included some unknown accessions and some local varieties provided by “Archeologia Arborea” (gr5). Cluster 2B included two commercial cultivars. Pera Briaca, Pera di Montelupone, Pera Monteleone and Pera Ruzza were assigned to Cluster 3B, characterized by winter ripening and a round fruit shape (Table S1). Cluster 4B included only commercial cultivars. Cluster 5B was represented by two local varieties (Pera Broccolina and Pera Volpina) and some cultivars, all characterized by autumn-winter ripening. On the contrary, Cluster 3B included accessions characterized by winter ripening and a round fruit shape (Supplementary Table S1). Cluster 6B included four local varieties and five reference species (*P. betulifolia, P. calleryana, P. caucasica, P. pyrifolia*, and *P. ussuriensis*).

### Genetic similarity

**Figure 2:**
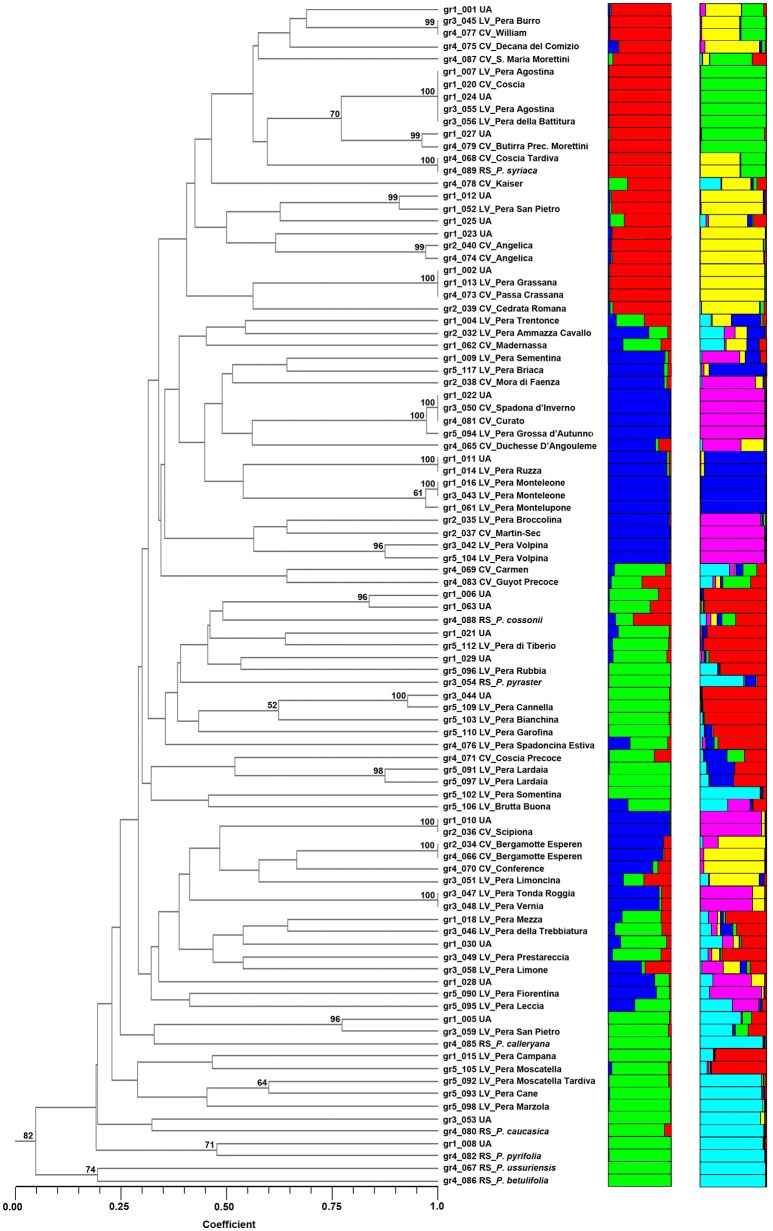
**The dendrogram of the 95 accessions of ***Pyrus*** spp. as clustered by UPGMA (on the left) and by STRUCTURE at ***K*** = 3 and ***K*** = 6 (on the right)**. Numbers on the cluster node indicate its probability (%) obtained by bootstrap.

The accessions of *Pyrus* spp. were clustered by UPGMA following the similarity estimates of Dice's coefficient (Figure 2). Reference species were found in a different subcluster, quite distant from most accessions, with the exception of *P. cossonii, P. pyraster*, and *P. syriaca*. In particular, *P. syriaca* is clustered with Coscia, Coscia Tardiva, Decana del Comizio, Santa Maria Morettini and William, while *P. cossonii* and *P. pyraster* with some local varieties, such as Pera di Tiberio and Pera Rubbia.

Several entries clustered at a similarity coefficient equal to 1. For example, the two accessions of Pera Agostina (gr1_007 and gr1_055) clustered with Coscia (gr1_020), Pera della Battitura (gr1_056) and one unknown accession (gr1_024), suggesting cases of synonymies with Coscia. As a matter of fact, “Agostina” in Italian means August, and “*battitura*” means “*threshing*,” most likely referring to the harvesting of cereals, normally occurring in summer, which is the ripening period of Coscia.

Similarly, Pera Grassana (gr1_013), Passa Crassana (gr4_073) and the unknown accession gr1_002 are all the same and likely to be Passa Crassana. Moreover, the present study confirmed that Spadona d'Inverno (gr3_050) and Curato (gr4_081) are synonyms and that gr1_022 (Unknown), clustering at a similarity of 1, is also likely to be the same accession. The same holds for Pera Tonda Roggia (gr3_047) and Pera Vernia (gr3_048), probably synonyms for the same genotype.

Furthermore, entry gr1_010 (Unknown) could be named Scipiona (gr2_036) and entry gr1_011 could be named Pera Ruzza (gr1_014). Entries gr1_016 and gr1_043, known as Pera Monteleone, are confirmed to have the same genotype and clustered together with Pera di Montelupone.

In addition to the clusters that joined at a similarity equal to 1, the analysis confirmed that the two accessions of Angelica clustered together, as well as the two accessions of Bergamotte Esperen and all the accessions of Pera Lardaia and Pera Volpina. The analysis summarized in Table S9 was also able to give insights for other accessions, such as Pera Grossa d'Autunno, similar to Curato and Spadona d'Inverno, and some unknown accessions that could be named as the closest commercial or local variety.

### CpDNA haplotype classification

The 95 novel chloroplast DNA sequences ranged from 622 to 899 bps depending on the presence of ten indels (four deletions at nps 210, 322, 545, and 601, and six insertions at nps 305, 544, 600, 620, 636, and 665); gr4_088 was the only accession without indels and differed from the reference sequence William only by the transversion at np 567 (567T). After grouping the accessions into four categories (reference species, RS; commercial varieties, CV; local varieties, LV; and unknown accessions, UA), AMOVA was carried out on all haplotypes and the results obtained through the SSR were confirmed. In fact, most of the observed variance was attributable to differences among samples within groups (96.69%), rather than the variability among groups (3.31%).

The analyses was then extended to the entire dataset of 298 cpDNA sequences (95 from the present study and 203 from GenBank). A total of 75 haplotypes were identified and named from HT01 to HT75 (Table S3). The 95 accessions of the present study were represented by 14 haplotypes, the most frequent of which was haplotype HT06 (47%). Out of these 14 haplotypes, 8 were shared among different accessions of *P. communis* (HT02, HT06, HT07, HT08, HT09, HT11, HT12, HT13), *P. caucasica* (HT09), *P. syriaca* (HT06) and some unknown accessions (HT02, HT06, HT08, HT09, HT12, HT13), while 6 were novel (Table S10): one was found in *P. ussuriensis* (HT18), one in *P. cossonii* (HT19) and the other four (HT08, HT11, HT12, and HT13) were found in *P. communis*. As evident in Figure S2, the new haplotypes (circled in red), whose sequences have never been reported before, were present in about 28% of the whole *P. communis* species and this value rises up to 50% if the most common haplotype HT06 is excluded from the analysis. This means that half of the *P. communis* samples have sequences never reported before. It is worth noting that four novel haplotypes (HT08, HT11, HT12, and HT13) were well represented in many accessions from Central Italy, most of which were provided by “Archeologia Arborea” (13 out of the 18 accessions, Table S1).

### CpDNA phylogenetic analyses

The reconstructed network of the cpDNA intergenic region *accD-psaI* clearly defined the distribution of the 95 accessions in different branches. All sequences clustered into two main groups, hereafter called Western and Eastern haplogroups (W and E, respectively, Figure S3) and were discriminated by the deletion of 20 bp at np 601. The Eastern group included only the four RS from Eastern Asia (*P. betulifolia, P. calleryana, P. pyrifolia*, and *P. ussuriensis*). The Western group included prevalently European and Mediterranean species, and encompassed all the unknown accessions (*N* = 20). These latter samples showed the two most common Western haplotypes, namely HT06 and HT09, encompassing different *Pyrus* spp., and five other haplotypes, four of them shared with accessions of *P. communis* (Figure S3).

**Figure 3:**
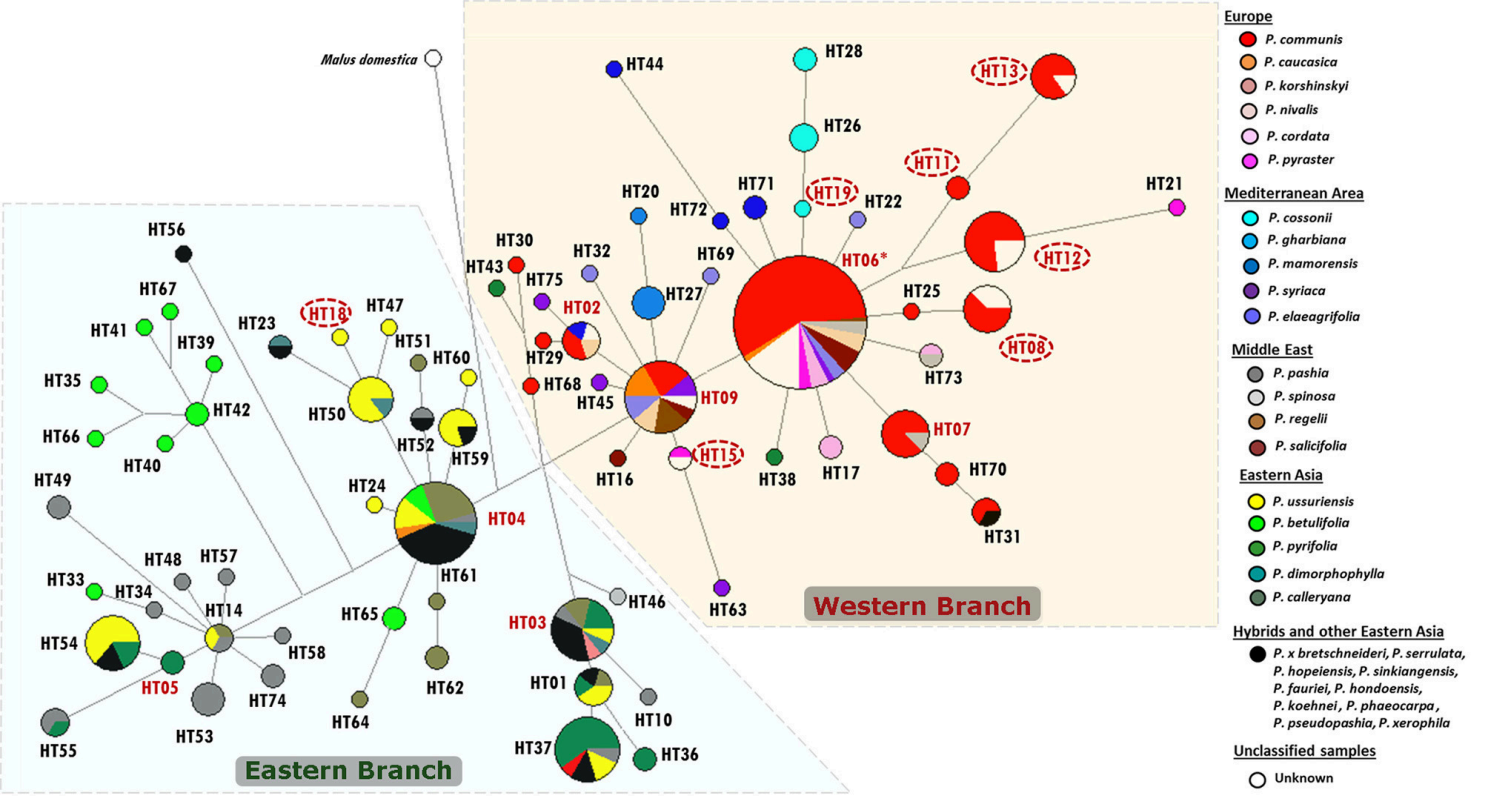
**Median-joining network based on all available ***accD-psaI*** sequences in the ***Pyrus*** genus**. Different species, each derived from specific geographic areas, are marked with different colors. Haplotype classification is detailed in Table S3 and Table S4. In particular, haplotypes derived from our samples are reported in red: circled when unique (see also Table S10), and not circled when shared with previously recorded sequences. The asterisk indicates the haplotype used as Reference Sequence.

A clear-cut geographic subdivision was also observed when all available sequences from GenBank were included in the network (Figure 3). The dominant haplotypes for the Western and Eastern geographic groups were HT06 (including the reference sequence) and HT04, respectively. Out of a total of 298 samples (95 from this study and 203 from GenBank), 169 accessions belonged to the Western haplogroup (57%), mostly derived from European species (61%), Mediterranean species (16%) and only a small amount from Eastern Asia (1.2% including only *P. pyrifolia*) and from the Middle East (*P. spinosa, P. salicifolia*, and *P. regelii*; 8.3%). These two latter species were absent in the Eastern haplogroup which presented only *P. pashia* (15.5%) and one sequence from *P. spinosa*, as representative of Middle Eastern species (Figure 3). In addition, the Eastern haplogroup is prevalently characterized by species from Eastern Asia (68.2%), no Mediterranean species, and only three sequences from European species (*P. caucasica, P. communis*, and *P. korshinskyi*).

The unknown accessions of the present study were included in the Western branch, and shared the same haplotypes of *P. communis* (HT08, HT12, and HT13), *P. pyraster* (HT15) and of other *Pyrus* spp. (HT02, HT06, and HT09).

**Figure 4:**
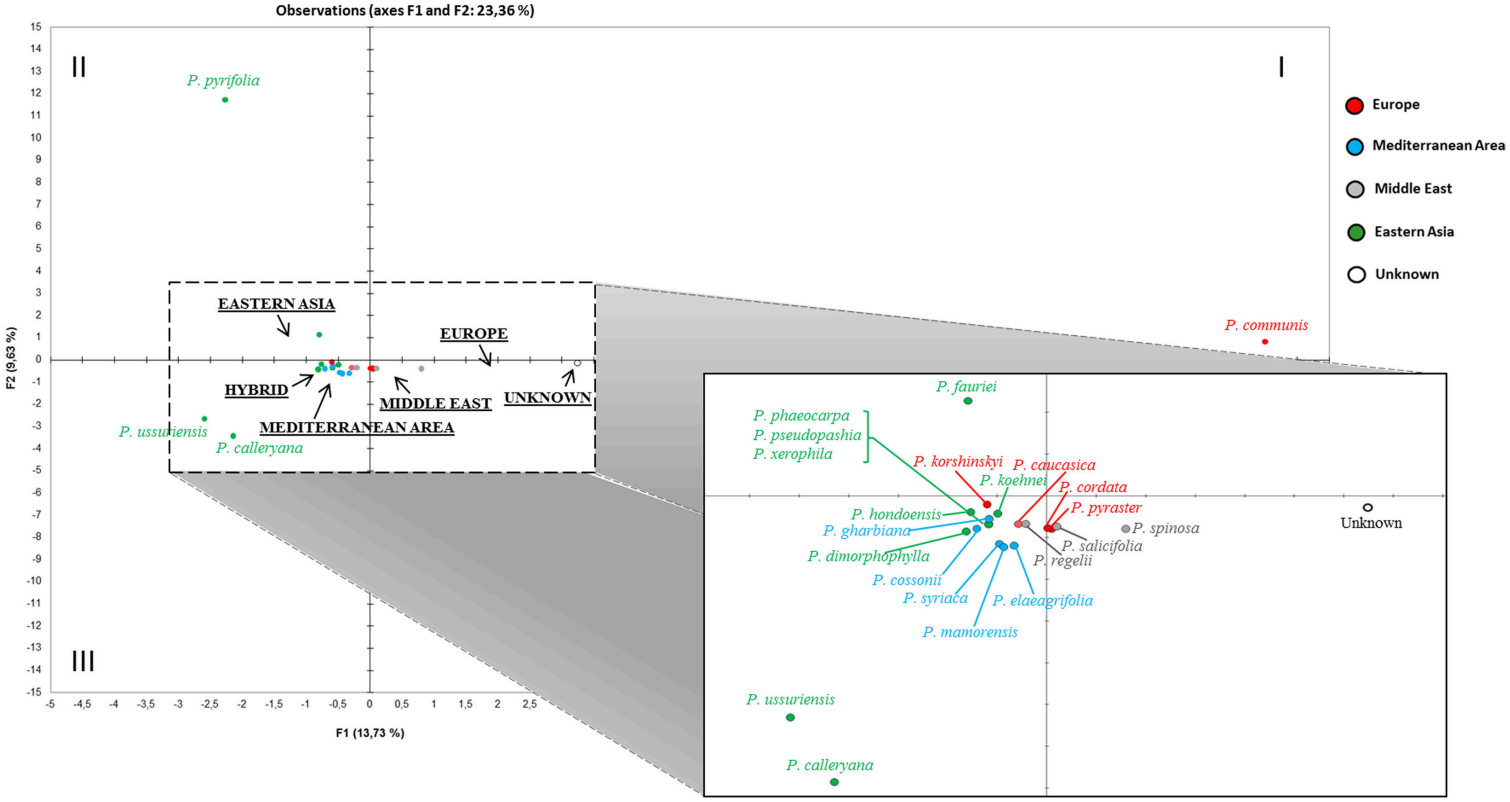
**Two-dimensional region-based PCA plot obtained by including all available ***Pyrus*** cpDNA data (see also Table S3 and Table S4)**. The most divergent *P. betulifolia* and *P. pashia*, and all hybrids were excluded from the final PC analysis. The macrogeographic areas are underlined and represent the centroids of species derived from the area. A magnification of the central mixed group is also presented.

The haplotype relationships were then summarized through principal component analysis (PCA) of the entire dataset. A preliminary PCA (not shown) was uninformative because of the distortion due to two evident outliers (*P*. *betulifolia* and *P*. *pashia*), both exclusively from the Eastern cluster in Figure 3, which moved far because of their unique haplotypes (HT33, HT35, HT39, HT40, HT41, HT42, HT65, HT66, HT67 for *P*. *betulifolia*, and HT10, HT34, HT48, HT49, HT53, HT57, HT58, HT74 for *P*. *pashia*). In order to better resolve the graph, a further analysis was performed without those two outliers (Figure 4). In the resulting PCA, some species split up into different quadrants: *P*. *communis* in the first, *P*. *pyrifolia* in the second, and *P*. *calleryana* and *P*. *ussuriensis* in the third one. The unknown accessions (grouped together here) were placed between *P. communis* and a mixed Western Eurasian group located in the center. Magnification of this group highlighted that the unknown accessions were nearby to a subgroup of four closely-related Middle Eastern (*P. salicifolia* and *P. spinosa*) and European (*P. cordata* and *P. pyraster*) species, which in turn were closer to *P. caucasica* and *P. regelii*, than to those from Eastern Asia and the Mediterranean area.

## Discussion

The possibility of identifying synonymous and homonymous accessions, sometimes even unknown genotypes, emphasized the importance of verifying germplasm collections with powerful tools such as molecular markers. This step is important in order to avoid redundancy in the collections, reduce their management costs and to be able to distribute true-to-type cultivars to nurseries. This was one of the goals of the present study, along with inference on the genetic structure and on the understanding of the geographic origin (phylogeny and evolutionary history) of pear accessions found in Central Italy. For both objectives reliable markers are essential for an accurate genetic identification, establishing genetic relationships among the accessions and improving the management and use of field-collected fruit tree germplasm.

The former task was partially achieved through nine SSR markers that displayed a high degree of polymorphism and discriminating power, a sufficient number to fingerprint germplasm collections (Urrestarazu et al., 2015). In summary, five accessions obtained from different sources were confirmed to be the same. Eight out of 20 accessions of unknown origin were identified. No homonymous accessions were present. Six synonyms were detected. In some instances, synonyms may be due to small changes in the names due to differences in local dialects (Pera Monteleone - Pera di Montelupone; Passa Crassana - Pera Grassana), and in other cases a variety may have been renamed to associate its ripening with some seasonal events (Coscia - Pera Agostina; Coscia - Pera della Battitura).

Understanding the genetic structure of pear accessions is more difficult to achieve than in most fruit tree species, as they are perennials and since ancient times their distribution has been generally human-mediated by clonal propagation (basically by grafting) in order to overcome the juvenile phase. The mode of reproduction, essentially outcrossing, and the human-mediated evolutionary processes (clonal propagation) played a critical role in the domestication and genetic variation found nowadays in most fruit tree species. For these species, domestication has been greatly affected by the propagation system, and studies in these fields, compared with annual species, are in progress. In evolutionary terms, by selecting and growing a high number of individuals with the same genotype (cultivar), a genetic bottleneck (Miller and Gross, 2011) has developed, which is expected to reduce the genetic variability of the species. Nevertheless, the selected genotype contains genetic variability in a potential state, conserved in the heterozygotic condition. Despite such observations, in perennial fruit tree crops the size of the genetic variation found within populations is generally significantly higher compared with that between populations, so that normally these populations are unstructured (Miller and Gross, 2011). High levels of heterozygosity and a high effective number of alleles per *locus* were found in the present study. By grouping the accessions into four categories (CV, LV, RS, and UA) the within group variation was as high as 97% compared with that among groups; even by grouping the genotypes by STRUCTURE the within variation was still as high as 87.5%, confirming a weak population structure (Loveless and Hamrick, 1984).

At *locus* NH030a, an F_ST_-value of 0.278 was found, indicating a high differentiation between RS and all other cultivated forms. Looking at the alleles in these two groups it was found that allele 167 was fixed in all individuals of CV, LV, UA compared with RS. In addition, among the RS, *P. caucasica, P. cossonii, P. pyraster*, and *P. syriaca* showed allele 167 fixed. This was partially confirmed by the cluster analysis, particularly for *P. cossonii* and *P. syriaca*, grouped together with many cultivated accessions, of which they are interfertile. Interestingly, *locus* NH030a was described as being associated with two QTLs, *Pfl-7-2* and *Pfi-7-1*, located on the 7th linkage group and involved in controlling fruit dimension (length) and fruit shape index, respectively (Zhang et al., 2013). This shed light on the SSR results and led to the hypothesis that allele fixation at this *locus* is most likely the result of human selection for fruit size. Moreover, this association is difficult to break if reproduction is carried out by clonal procedures.

Among the 95 accessions, 52 were diploids and 43 (45%) were putative polyploids, since at least 3 alleles were found in at least 1 of the 9 *loci*. Reference species were generally diploids, except for *P. pyrifolia* that is characterized by 5 *loci* with three alleles. But, as pointed out by Ferreira dos Santos et al. (2011), the amplification of three fragments in a single *locus* is not proof of the triploid status: mutational events (somatic mutations generating chimerical or mosaic states, or duplication events of a chromosome fragment) might give rise to non-real alleles. In our case, considering only individuals showing at least 2 or more loci with a third allele, the percentage of putative polyploids is 20%, a value similar to 18% reported by Ferreira dos Santos et al. (2011).

The uniparental genetic analysis confirmed the AMOVA on SSRs, with the size of the within group variability enormously greater than that among groups (96.7 vs. 3.3%, respectively).

Based on the available phylogenetic data, the large number of varieties of the cultivated European pears is most likely derived from one or two wild species (*P. pyraster* and/or *P. caucasica*), widely distributed throughout Europe (Vavilov, 1992). The analysis on the intergenic segment *accD-psaI* supported the opinion of a clear division between Occidental and Oriental species. The network analysis of 298 cpDNA sequences allowed two different haplogroups with a geographic prevalence in Eastern and Western Eurasia to be defined. The sequences of *P. caucasica* and *P. pyraster* presented mostly western haplotypes (HT06, HT09, HT15, and HT21); an eastern haplotype was identified only in *P. caucasica* (HT04). HT06 and HT09 were the most frequent and variable in the Western haplogroup and presented other different species belonging to all the geographic areas, except for Eastern Asia. Remarkably, one of our unknown accessions shared haplotype HT15 with an accession of *P. pyraster*, indicating a possible connection between them, confirmed also by their inclusion in the same STRUCTURE Cluster (Figures 1, 2A). Furthermore, haplotype HT21 (present only in *P. pyraster* and absent in all our 95 accessions) is closely related to HT12, one of the novel haplotypes identified here. HT12 includes ten accessions of *P. communis* (all LV, mostly from Archeologia Arborea) and three unknown accessions from Central Italy; the proximity of HT21 and HT12 suggests an ancient origin of them. In addition, haplotype HT12 and HT21 were closely clustered to HT11 and HT13, all represented by local varieties, again mostly from Archeologia Arborea, and further highlighting their possible ancient origin.

By combining the results from the SSRs and cpDNA, it was possible to better define many unknown accessions. For instance, HT07 included almost all local varieties (86%), two of them (Pera Tonda Roggia and Pera Vernia) were found as synonyms according to the SSR results. Haplotype HT08 included a total of eight accessions, four commercial varieties, one local variety and three unknown accessions. These unknown accessions could be classified as *P. communis*. Moreover, one of them (gr1_010) showed the same haplotype as Scipiona, which is a further validation of the similarity found by SSR, so that it might consider as the same genotype. Sharing the same haplotype (HT08) also confirmed that Curato and Spadona d'Inverno could be considered synonyms, while Pera Grossa d'Autunno, considered to be Spadona d'Inverno by the SSR profile, showed a different haplotype (HT11) and needs to be reconsidered.

HT12 included 13 accessions, ten local varieties and three unknown accessions. Therefore, the unknowns are expected to be *P. communis*; one of them (gr1_011) was confirmed to be Pera Ruzza, while gr1_44 was confirmed to be Pera Cannella. The same haplotype also included the two accessions of Pera Lardaia, the two accessions of Pera Monteleone and Pera di Montelupone, confirming once again the SSR results.

HT13 included only one unknown accession (gr1_025) and this could be ascribed to *P. communis*.

All together out of the 20 unknown accessions, seven could be classified at least at a species level, namely as *P. communis* (4 CV and 3 LV). These results confirm the usefulness of comparing the two molecular marker systems for evaluating the genetic variability of local and unknown accessions. The uniparental genetic system leads to reconstructing the phylogenetic relationships of different cultivars and species. The comparison between the haplotypes found in our accessions and that from GenBank highlighted that 99% of the accessions of *P. communis*, all Mediterranean accessions and all European accessions, *P. cordata, P. nivalis*, and *P. pyraster*, belonged to the Western clade, which also included some sequences with a Middle Eastern origin, but without any *P. pashia* representatives. This result also strengthens the hypothesis of the proximity of European pears to some species of Mediterranean and Middle Eastern origin.

## Author contributions

EA and FV conceived the study; EA designed and coordinated the experiments; IR, MG, and LC chose and provided the germplasm; IR provided the historical and anthropological information; NF and GM performed the lab experiments; NF, RT and LR conducted the SSR data analysis; AA coordinated the analysis on the cpDNA; HL and IC performed the analysis on the cpDNA; NF, HL, RT, LR, IC, and EA wrote the manuscript; NF, HL, RT, LR, IC, GM, AA, and EA revised the manuscript.

## Funding

The research was supported by “Convenzione con il Parco 3APTA 2014: servizio di conservazione e ampliamento delle banche regionali della biodiversità,” by the personal funding of Prof. Albertini (ALBPRESTAZ and ALBRICVAR) and by the Italian Ministry of Education, University and Research: Progetti Futuro in Ricerca 2012 (RBFR126B8I).

### Conflict of interest statement

The authors declare that the research was conducted in the absence of any commercial or financial relationships that could be construed as a potential conflict of interest.
