# Supplementary figures and images for: Characterization and Phylogenetic Analysis of Ancient Italian Landraces of Pear
Source: Front Plant Sci. 2017 May 10;8:751. doi: 10.3389/fpls.2017.00751 (PMC5423897; doi:10.3389/fpls.2017.00751)

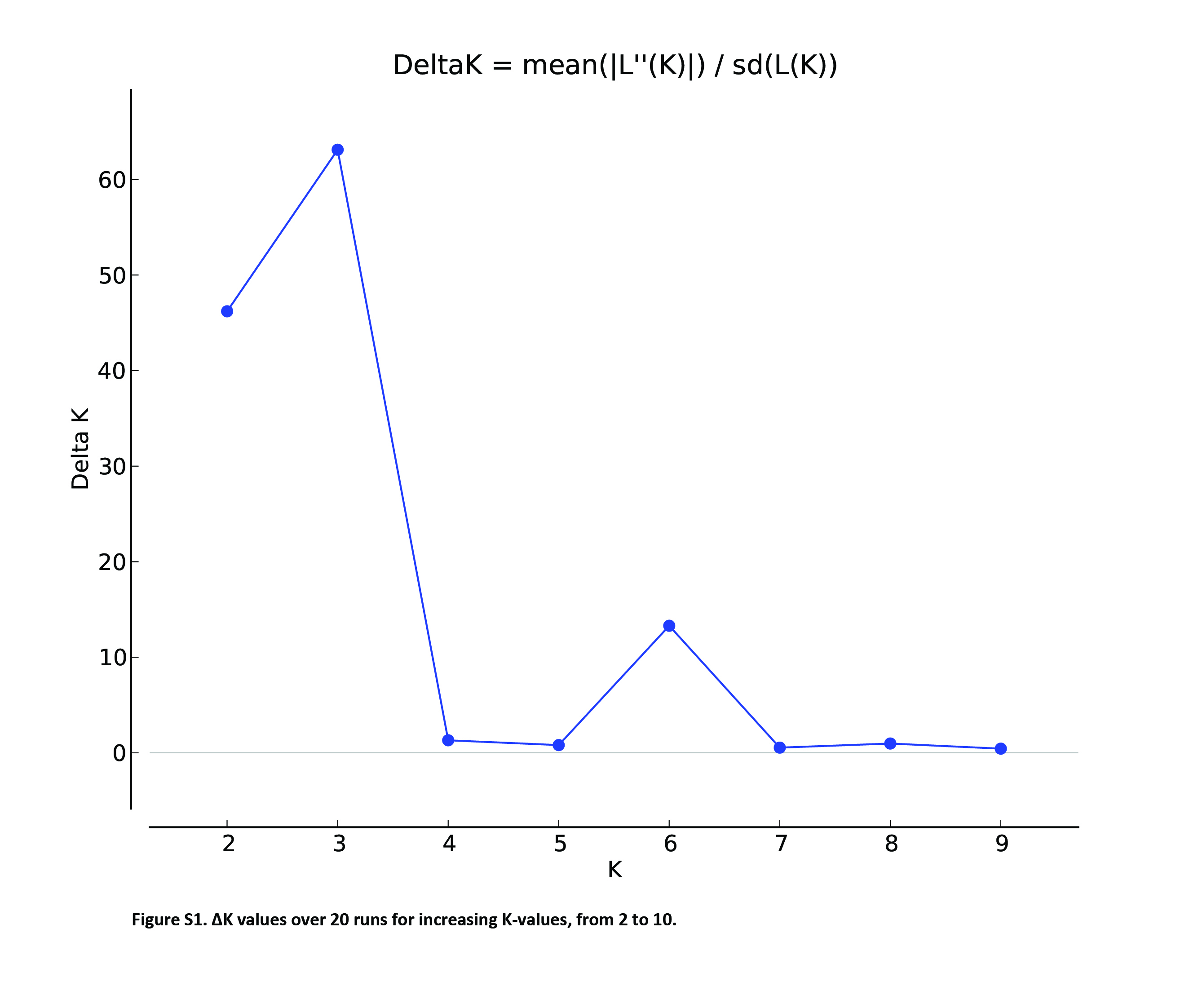

Supplement: Supplementary file 11 [file Image1.JPEG]

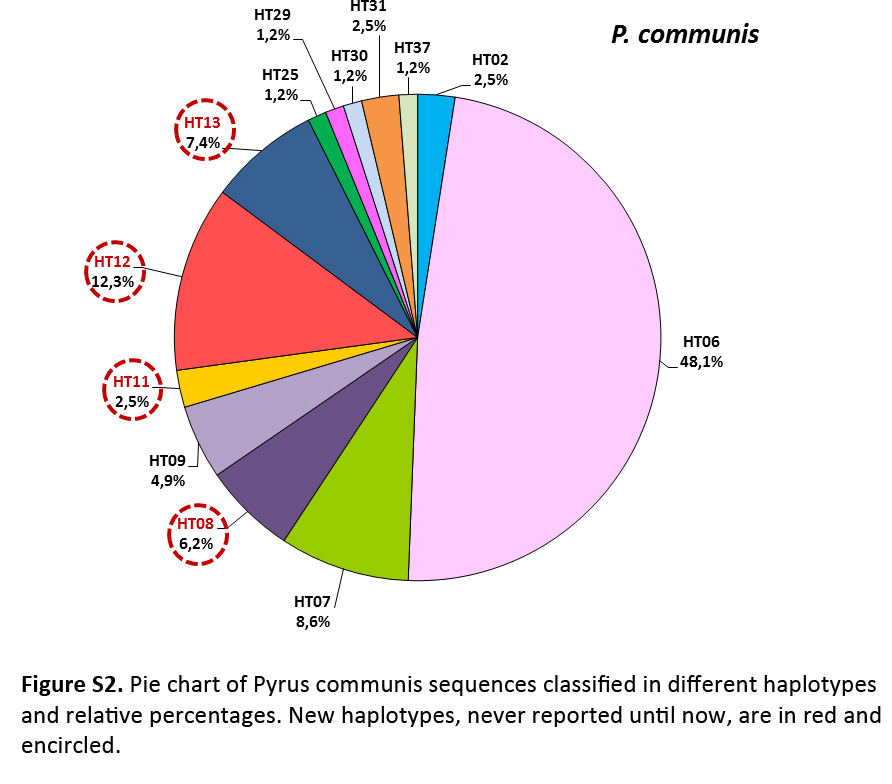

Supplement: Supplementary file 12 [file Image2.JPEG]

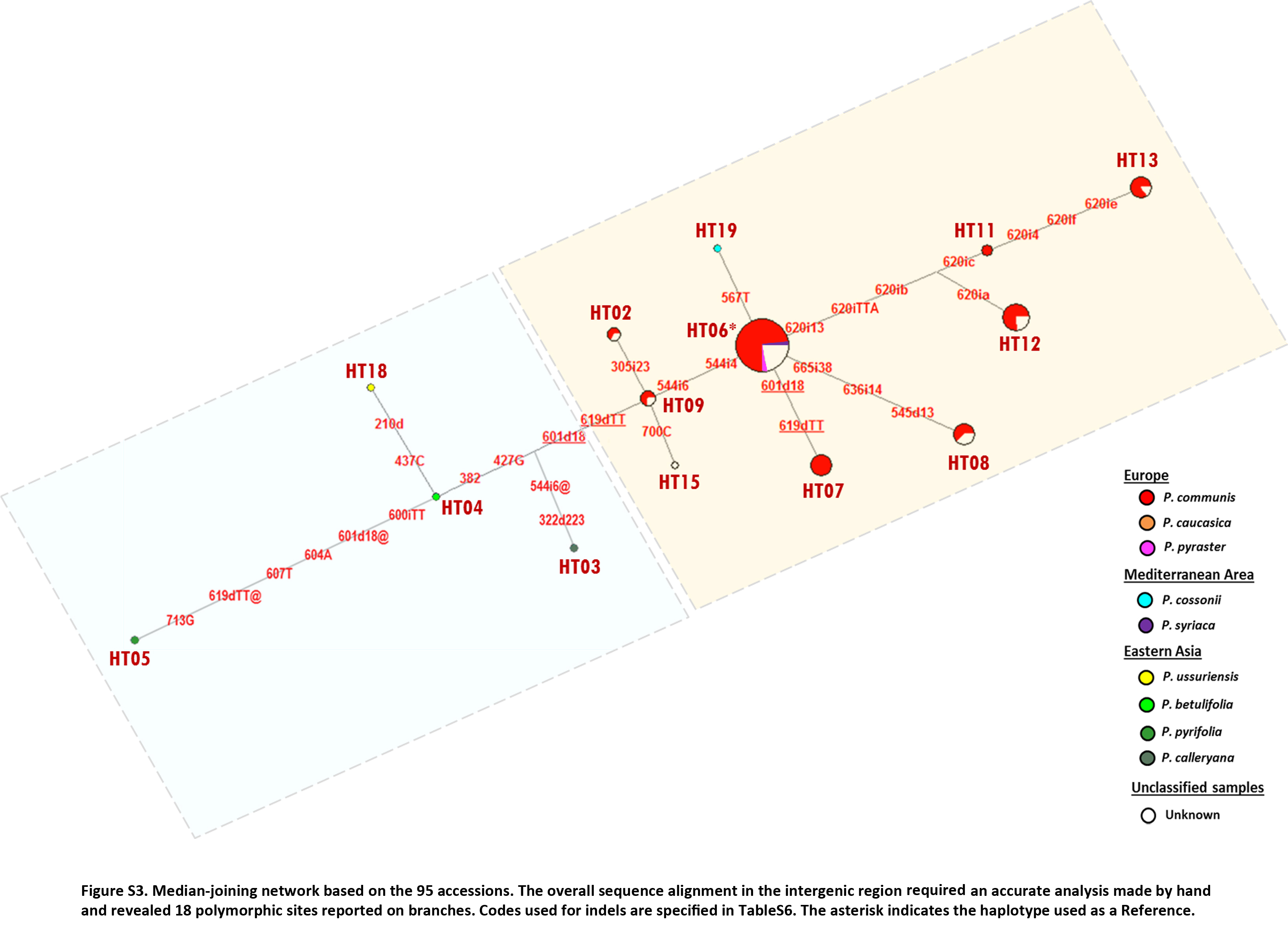

Supplement: Supplementary file 13 [file Image3.JPEG]
